# P2A-mediated cotranslation bypasses GESENI, a cryptic gene silencing system in *Arabidopsis* sperm cells

**DOI:** 10.1093/pcp/pcag014

**Published:** 2026-02-04

**Authors:** Daigo Ishida, Naoya Sugi, Kazuki Motomura, Daichi Susaki, Daisuke Maruyama

**Affiliations:** Kihara Institute for Biological Research, Yokohama City University, Yokohama, 244-0813, Japan; Kihara Institute for Biological Research, Yokohama City University, Yokohama, 244-0813, Japan; Laboratoire Reproduction et Développement des Plantes, Univ Lyon, ENS de Lyon, UCB Lyon 1, CNRS, INRAE, Lyon 69342, France; Research Organization of Science and Technology, Ritsumeikan University, Kusatsu, 525-8577, Japan; Kihara Institute for Biological Research, Yokohama City University, Yokohama, 244-0813, Japan; Department of Biological Science, Faculty of Science, Shizuoka University, Shizuoka 422-8529, Japan; Department of Science, Graduate School of Integrated Science and Technology, Shizuoka University, Shizuoka 422-8529, Japan; Kihara Institute for Biological Research, Yokohama City University, Yokohama, 244-0813, Japan

**Keywords:** *Arabidopsis thaliana*, GESENI, ribosomal skipping, sperm cells

During double fertilization, two sperm cells are delivered through the pollen tube to the egg cell and central cell, and fusion of these two pairs of gametes results in the formation of the embryo and endosperm (Zhong et al. 2025). In *Arabidopsis thaliana*, gamete fusion occurs rapidly, completing about 7 min after pollen tube rupture (Hamamura et al. 2011). Despite their importance in double fertilization, many aspects of sperm cell behavior and the molecular mechanisms involved remain unresolved. To our knowledge, calcium dynamics in sperm cells before or after double fertilization have not been well characterized, partly due to an enigmatic gene silencing phenomenon in sperm cells termed “GESENI” [gesénài] (GEne Silencing based on ENcoded protein’s Intracellular localization). This term means “incomprehensible” in Japanese (Ohnishi and Kawashima 2023). Here, we describe a strategy that enables expression of transgenes susceptible to GESENI.

To monitor cytosolic calcium levels in sperm cells, we generated transgenic plants carrying the coding sequence of the GCaMP calcium biosensor downstream of the sperm cell–specific *HISTONE THREE RELATED 10* (*HTR10*) promoter (Supplementary Material). In 20 independent T1 plants, mature pollen showed no detectable GCaMP fluorescence, similar to nontransgenic wild-type plants (Fig. 1a and b and Supplementary Table S1). A GCaMP variant previously used for calcium imaging of the pollen tube vegetative cell was the same as that employed in this study (designated hereafter “GCaMPno”) (Sugi et al. 2024a). Thus, the lack of GCaMP expression was unlikely due to incompatibility of GCaMPno with the *Arabidopsis* male gametophyte. We performed codon optimization of GCaMP and attempted expression under either the *HTR10* promoter or another sperm cell-specific promoter, *DUO POLLEN 1* (*DUO1*). However, these approaches produced no detectable signals (Fig. 1c and d). Our GCaMP constructs met three GESENI-inducible criteria: they are transgenes, encode a cytosolic protein, and are regulated by sperm cell–active promoters (Ohnishi and Kawashima 2023). Notably, none of the pollen grains from these lines exhibited even weak fluorescent signals (Fig. 1b–d, Supplementary Table S1), although pollen harboring several GESENI-targeted constructs has been reported to produce weak fluorescence at low frequency (Ohnishi and Kawashima 2023).

**Figure 1:**
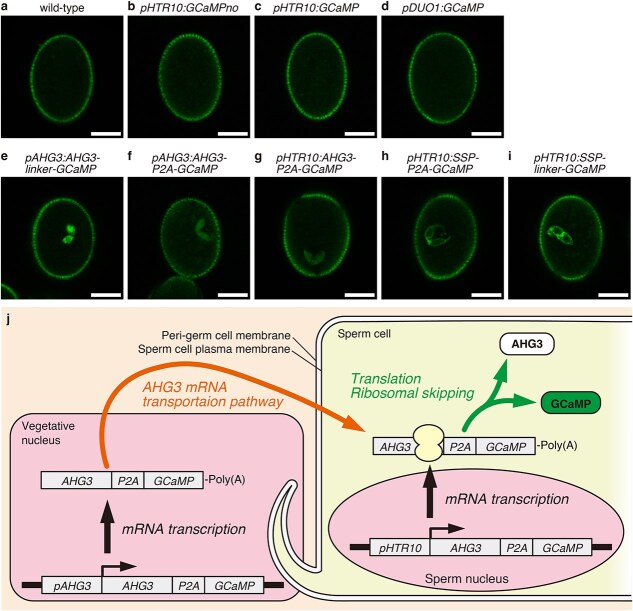
Bypassing sperm cell–specific gene silencing GESENI. Representative confocal images of mature pollen grains are shown for (a) wild-type, (b) *pHTR10:GCaMPno*, (c) *pHTR10:GCaMP*, (d) *pDUO1:GCaMP*, (e) *pAHG3:AHG3-*linker*-GCaMP*, (f) *pAHG3:AHG3-P2A-GCaMP*, (g) *pHTR10:AHG3-P2A-GCaMP*, (h) *pHTR10:SSP-P2A-GCaMP*, and (i) *pHTR10:SSP*-linker-*GCaMP*. The fluorescence observed around the pollen surface represents autofluorescence. (j) Schematic diagram of the proposed GESENI bypassing pathways. All the confocal images were acquired using identical microscope settings. Scale bars: 10 μm.

GESENI involves transcriptional or posttranscriptional reduction of mRNA from targeted genes. To circumvent GESENI, we employed an intercellular mRNA transport pathway. Transcripts of *ABA-HYPERSENSITIVE GERMINATION3* (*AHG3*) are produced in the vegetative cell but translated in the sperm cells, presumably after passing two membrane barriers: the peri-germ cell membrane and the sperm cell plasma membrane (Jiang et al. 2015, Sugi et al. 2024b). An *AHG3* translational fusion reporter of GCaMP could label sperm cells; however, the signal was mainly localized to the nuclei, as reported previously (Fig. 1e). To achieve cytosolic localization of GCaMP and remove the bulky AHG3 protein, we used P2A, a short peptide tag that generates independent proteins from a single mRNA via ribosomal skipping during translation (El Amrani et al. 2004). By introducing an *AHG3* translational fusion reporter gene containing P2A instead of a linker between AHG3 and GCaMP, we obtained transgenic lines that exhibited uniformly diffused GCaMP signals in sperm cell cytosol (5 out of 10 independent T1 lines; Fig. 1f and Supplementary Table S2). Although fluorescence occurred at a frequency lower than the expected ratio, these constructs partially bypassed GESENI and enabled pollen to produce sufficiently strong fluorescent signals (Fig. 1f and Supplementary Table S2). We next tested whether P2A-mediated cytosolic GCaMP production was also applicable in transformants expressing AHG3-P2A-GCaMP from the *HTR10* promoter. A considerable proportion of T1 plants displayed cytosolic GCaMP signals, indicating successful bypass of GESENI, presumably following direct mRNA transcription in the sperm cells (Fig. 1g). However, P2A-mediated ribosomal skipping may not apply to all noncytosolic proteins. When the sperm cell plasma membrane–localized protein SHORT SUSPENSOR/BRASSINOSTEROID SIGNALING KINASE 12 (hereafter “SSP”), previously identified as a gene not targeted by GESENI (Ohnishi and Kawashima 2023), was fused to P2A-GCaMP, fluorescence was detected in the sperm cell plasma membrane similar to that of SSP-linker-GCaMP (Fig. 1h and i). The ribosomal skipping efficiency mediated by P2A is inherently imperfect and can be affected by the flanking sequences (El Amrani et al. 2004). Therefore, in the case of SSP-P2A-GCaMP, the skipping efficiency may have been insufficient. Despite possible incompatibility between fusion partners, ribosomal skipping remains an effective method to bypass GESENI, and transcript delivery from the vegetative cell is also feasible (Fig. 1j). Furthermore, we confirmed that the strategy is effective with fluorescent proteins other than GCaMP (Supplementary Fig. S1, Supplementary Tables S1 and S2).

Our study demonstrated that cytosolic proteins can bypass GESENI through the P2A sequence, although the underlying mechanism remains unknown. Further work is required to identify the genuine silencing signal of transgenes that distinguishes them from endogenous cytosolic protein genes in sperm cells. GESENI may have acted as an overlooked barrier in analyzing sperm cells using genetic engineering tools such as biosensors. For instance, genetically encoded calcium biosensors, including Yellow Chameleon 3.60, CerTN-L15, R-GECO1, and GCaMP, have advanced plant reproduction research by enabling visualization of calcium dynamics in egg cells, central cells, synergid cells, and pollen vegetative cells (Denninger et al. 2014, Hamamura et al. 2014, Ngo et al. 2014, Sugi et al. 2024a). However, calcium dynamics in sperm cells have not yet been characterized. Our GCaMP-expressing transgenic plants offer an unprecedented tool to visualize calcium signaling in sperm cells. Beyond biosensor expression, the GESENI bypass strategy opens the door to new experiments requiring functional cytosolic proteins. Eliminating gene expression barriers in sperm cells for metabolic enzymes, kinases/phosphatases, proteases, and genome editing tools could further advance research on double fertilization and breeding in flowering plants.

## Supplementary Material

Supplementary_Data_pcag014

Supplementary_Tables_S1_and_S2_pcag014

## Data Availability

All the data are available upon request to the corresponding author.
