# Supplementary material for: P2A-mediated cotranslation bypasses GESENI, a cryptic gene silencing system in Arabidopsis sperm cells
Source: Plant Cell Physiol. 2026 Feb 4;67(6):866–8. doi: 10.1093/pcp/pcag014 (PMC13317974; doi:10.1093/pcp/pcag014)
Supplement: Supplementary_Data_pcag014 [file supplementary_data_pcag014.docx]

# *Supplementary Data*

**P2A-mediated Co-translation Bypasses GESENI, a Cryptic Gene Silencing System in Arabidopsis Sperm Cells**

**Daigo Ishida, Naoya Sugi*, Kazuki Motomura, Daichi Susaki, Daisuke Maruyama**

***Correspondence:** Naoya Sugi: sugi.nao.qm@yokohama-cu.ac.jp

# **Materials and Methods**

*Plant material and Growth condition*

*Arabidopsis thaliana* Columbia-0 (Col-0) served as the WT plant. Seeds were germinated on Murashige-Skoog medium and subsequently transferred to soil. The plants were cultivated at 22°C under continuous lighting conditions.

*Plasmid construction*

The *GCaMP6f* original sequence, derived from the pGP-CMV-GCaMP6f (#40755, addgene) (Chen et al., 2013), was amplified from *pLAT52:GCaMP* (Sugi et al. 2024) using attB1-GCaMP_F (5’-GGG GAC AAG TTT GTA CAA AAA AGC AGG CTA TGG TCG ACT CAT CAC GTC G-3’) and GCaMP-attB2_R (5’-GGG GAC CAC TTT GTA CAA GAA AGC TGG GTT CAC TTC GCT GTC ATC ATT TG-3’) as primers, and the *GCaMP* DNA fragment was cloned into the pDONR221 (Thermo Fisher Scientific, USA) by a BP recombination using the BP clonase II enzyme mix (Thermo Fisher Scientific, USA) to produce pSG41. The pSG50, a pDONR221 vector containing the codon-optimized *GCaMP6f* sequence, was synthesized by Thermo Fisher Scientific. The pSG41 or pSG50 were mixed with a pGWB501 destination vector carrying the *HTR10* promoter designated pDM252 (Motomura et al., 2021) and subjected to LR recombination using the LR clonase II enzyme mix (Thermo Fisher Scientific, USA) to produce pSG65 or pSG52, respectively.

To generate a plasmid containing the *pDUO1:GCAaMP*, 1,312 bp upstream sequence of start codon of the DUO1 was amplified from *Arabidopsis* Col-0 genomic DNA using DUO1pro_HindIII_F (5’-CGC AAG CTT TGT TCT GGA AGT TTG TTG TTG-3’) and DUO1pro_HindIII_R (5’-GCG AAG CTT TCC TCA TCG CTA ATC GAT CTC-3’) as primers. The *DUO1* promoter DNA fragment was digested with HindIII and inserted into HindIII site of the pGWB501 (Nakagawa et al., 2007) to produce pDM539. An LR recombination between the pDM539 and pSG50 produced pSG53 expression vector carrying the *pDUO1:GCAaMP*.

To obtain an entry clone of *SSP-P2A-GCaMP*, pSG50 was amplified by PCR using the primers P2A_GCaMP_F (5’- GCT CAA GCA GGC CGG CGA CGT GGA AGA GAA TCC CGG CCC CAT GGT GGA CTC TTC TAG GCG -3’) and GCaMPopt-attB2_R (5’- GGG GAC CAC TTT GTA CAA GAA AGC TGG GTT CAC TTG GCG GTC ATC ATC -3’) to generate the P2A-GCaMP fragment. Simultaneously, *Arabidopsis* Col-0 genomic DNA was amplified by PCR using attB1_SSP_F (5’- GGG GAC AAG TTT GTA CAA AAA AGC AGG CTA TGG GTT GTT GTT ACT CAC TAT C -3’) and P2A_SSP_R (5’- CGT CGC CGG CCT GCT TGA GCA GGG AAA AGT TGG TGG CTT TCT TTA CTT CAA GAA GAG CTG C -3’) to obtain the SSP-P2A fragment. These two fragments were assembled using NEBuilder® HiFi DNA Assembly Master Mix (New England Biolabs, USA), and the resulting product was used in a BP recombination reaction with pDONR221 to generate entry clone pSG56. Finally, pSG56 was mixed with pDM252 and subjected to LR recombination using the LR Clonase II enzyme mix (Thermo Fisher Scientific, USA) to produce pSG59, an expression vector carrying *pHTR10:SSP-P2A-GCaMP*.

To substitute GS linker with P2A, pSG59 was linearized by a PCR using GCaMPQC2_F and SSPQC2_R (5’-ACC TCC AGA TCC ACC ACC TCC AGA GGA TTT CTT TAC TTC AAG AAG AGC TGC TTC TTT C-3’) as primers. Subsequent transformation of the PCR fragment after DpnI digestion produced pSG67, an expression vector carrying the *pHTR10:SSP-Linker-GCaMP*.

To generate a plasmid containing *pAHG3:AHG3-Clover-AHG3ter*, an intermediate vector was first constructed. A *Clover* fragment was amplified by PCR from a synthesized mClover sequence using MUp1732 (5'-CTA CTA CTA ATT ATA ACA CTA ATT ATT ACT TGT ACA GCT CGT CCA-3') and MUp1736 (5'-TTC AAG GGC GAA TTC AAT TGG GAT CCT CTG GAG GTG GTG GAT CTG-3') as primers. The *AHG3* terminator was amplified from Arabidopsis Col-0 genomic DNA using MUp1733 (5'-TAA TTA GTG TTA TAA TTA GTA GTA GTA TTA-3') and MUp1737 (5'-ACG CAT AAT CTG TTC TTT CAT TTT AAC-3') as primers. These two fragments and a vector backbone, which was amplified from the plasmid MU1937 (Kimata et al. 2016) with primers MUp1738 (5'-TTA AAA TGA AAG AAC AGA TTA TGC GTC TAG AAG GCG CGC CAT TAA-3') and MUp1739 (5'-CAA TTG AAT TCG CCC TTG AAA C-3'), were assembled using the Gibson Assembly Master Mix (NEB, USA). Subsequently, a DNA fragment containing the *AHG3* promoter (a 2,120 bp sequence upstream of the start codon) and the AHG3 ORF without the stop codon was amplified from Arabidopsis Col-0 genomic DNA using KMol123 (5'-TTG GTT TCA AGG GCG AAT TCA ATT GGA AGT GAA ACC AAG CAC AAG-3') and KMol057 (5'-CCT CCA GAT CCA CCA CCT CCA GAG GAA GAC GAC GCT TGA TTA TTC-3') as primers. This PCR product was inserted into the BamHI site upstream of the Clover sequence of the intermediate vector by Gibson Assembly to produce KMd104, an expression vector carrying *pAHG3:AHG3-Clover-AHG3ter*.

To substitute the GS linker-Clover with P2A-GCaMP, the SacII-digested KMd104 was amplified by PCR using the primers GCaMP-AHG3ter_F (5’- CAG ATG ATG ACC GCC AAG TGA TAA TTA GTG TTA TAA TTA GTA G -3’) and AHG3-P2A_R (5’- AGC AGG GAA AAG TTG GTG GCA GAC GAC GCT TGA TTA TTC C -3’) to generate a fragment of *pAHG3:AHG3-P2A* along with the backbone vector. Simultaneously, pSG56 was amplified by PCR using the primers P2A_F (5’- GCC ACC AAC TTT TCC CTG CTC -3’) and GCaMPpot_R (5’- TCA CTT GGC GGT CAT CAT CTG -3’) to produce the *P2A-GCaMP* fragment. These two fragments were assembled using NEBuilder® HiFi DNA Assembly Master Mix (New England Biolabs, USA) to construct pSG58, an expression vector carrying *pAHG3:AHG3-P2A-GCaMP*.

To substitute GS linker with P2A, pSG66 was linearized by a PCR using GCaMPQC2_F (5’-TGG TGG ATC TGG AGG TGG TGG AGC CAT GGT GGA CTC TTC TAG GCG TAA GTG GAA CAA G-3’) and AHG3QC_R (5’-ACC TCC AGA TCC ACC ACC TCC AGA GGA AGA CGA CGC TTG ATT ATT CCT C-3’) as primers. Subsequent transformation of the PCR fragment after DpnI digestion produced pSG66, an expression vector carrying the *pAHG3:AHG3-Linker-GCaMP-AHG3ter*.

To obtain *AHG3-P2A-GCaMP* expressed from the *HTR10* promoter, we amplified the *AHG3-P2A-GCaMP* from the pSG58 by using attB1-AHG3_F (5’-GGG GAC AAG TTT GTA CAA AAA AGC AGG CTA TGG CTG GGA TTT GTT GCG G-3’) and GCaMPopt-attB2_R (5’-GGG GAC CAC TTT GTA CAA GAA AGC TGG GTT CAC TTG GCG GTC ATC ATC-3’) as primers, and the PCR product was cloned into the pDONR221 by a BP recombination to produce pSG41, an entry clone carrying the *AHG3-P2A-GCaMP* sequence. An LR recombination between the pSG41 and pDM252 produced pSG64 expression vector carrying the *pHTR10:AHG3-P2A-GCaMP*.

To generate a plasmid containing *pAHG3:AHG3-P2A-mSG*, we first prepared an entry clone for mStayGold. A codon-optimized mStayGold variant mStayGold(QC2-6 FIQ(c4)) was synthesized de novo and cloned into the Gateway pDONR221 vector to generate pDS241 (Ando et al., 2023). From the pDS241, 738 bp of *mSG* was amplified by a PCR using mSG_pSG058_F (5’- GCC GGC GAC GTG GAA GAG AAT CCC GGC CCC ATG GTT TCA ACA GGC GAA G -3’) and mSG_pSG058_R (5’- TAC TAC TAC TAA TTA TAA CAC TAA TTA TCA AAG ATG AGC TTC CAG AGT TTC ACT CTG -3’) as primers. A binary vector harboring the *pAHG3:AHG3-P2A* with the total length 14,426 bp was amplified from the pSG58 by an inverse PCR using pSG058_backbone_F (5’- TGA　TAA　TTA　GTG　TTA　TAA　TTA　GTA　GTA　GTA　TTA　GAA　TAA　TTG -3’) and mSG_pSG058_R (5’- GGG　GCC　GGG　ATT　CTC　TTC　CAC -3’) as primers. These two fragments were assembled using NEBuilder® HiFi DNA Assembly Master Mix (New England Biolabs, USA) to construct pDM863, an expression vector carrying *pAHG3:AHG3-P2A-mSG*.

To generate a plasmid containing *pAHG3:AHG3-P2A-mSc3H*, a pDONR221 entry clone containing codon-optimized *mScarlet3-H* (*mSc3H*) designated pOR180 was purchased from Thermo Fisher Scientific (Xiong et al. 2025). Then, 738 bp of *mSG* fragment or 747 bp of *mSc3H* fragment were amplified from the above entry clones using mSc3H_pSG058_F (5’- GCC GGC GAC GTG GAA GAG AAT CCC GGC CCC ATG GAT TCT ACC GAG GCC G -3’) and mSc3H_pSG058_R (5’- TAC TAC TAC TAA TTA TAA CAC TAA TTA TCA AGA TCC TCC GCT TCC ACC -3’) as primers. The *mSc3H* fragment was introduce into the *pAHG3:AHG3-P2A* binary vector backbone used in the pDM863 construction above by the NEBuilder® HiFi DNA Assembly Master Mix (New England Biolabs, USA) to construct pDM880, an expression vector carrying *pAHG3:AHG3-P2A-mSc3H*.

To generate a plasmid containing *pHTR10:HTR10-tdSG*, we first synthesized a codon-optimized tandem dimer StayGold cassette, (n1)StayGold(c4)–EVlinker–(n1)StayGold, and inserted it into the SmaI site of the pPZP211 binary vector to obtain pDS180 (Hirano et al., 2022). Then, the pDS180 backbone carrying the *tdSG* was amplified by an inverse PCR using pDS180linearize_F (5’- GGT GGT TCT GGT GGA TCT GGA GGA TCT GGA ATG GCT TCT ACT GGA GAG GAG CTC TTT ACC GGA GTT G-3’) and pDS180linearize_R (5’- GGG GAT CCT CTA GAG TCG ACC TGC AGG CAT G-3’). The *pHTR10:HTR10* sequence including 1,215 bp upstream sequence of start codon of the *HTR10* was amplified from *Arabidopsis* Col-0 genomic DNA using pDS180_pHTR10_F (5’- ATG CCT GCA GGT CGA CTC TAG AGG ATC CCC TAC TTC TCC GAC CAA AAA CTT TC-3’) and pDS180_pHTR10_R (5’- TCC AGA TCC TCC AGA TCC ACC AGA ACC ACC AGC ACG TTC CCC ACG AAT G-3’) as primers. The *pHTR10:HTR10* sequence (2,031 bp) and linearized pDS180 (11,038 bp) were assembled using NEBuilder® HiFi DNA Assembly Master Mix (New England Biolabs, USA) to construct pDM869, an expression vector carrying *pHTR10:HTR10-tdSG*.

*Confocal imaging of mature pollen grains*

Mature pollen grains were mounted on pollen tube growth medium (0.01% (w/v) boric acid, 5 mM CaCl_2_, 5 mM KCl, 1 mM MgSO_4_, 10% (w/v) sucrose) and gently cover slipped. Confocal images were obtained using a Leica SP8 TCS equipped with a white light laser and three hybrid detectors (Leica, Wetzlar, Germany). We used 63× glycerol-immersion objective lens and 491 nm laser irradiation for observation, and the fluorescent signals were detected by the photon counting mode using a hybrid detector. Pollen from T1 plants was used to obtain the images shown in Figure 1. As exceptions, pollen from T2 plants exhibiting antibiotic resistance was used for samples *pHTR10:GCaMPno*, *pHTR10:GCaMP*, *pDUO1:GCaMP*, and *pAHG3:AHG3-linker-GCaMP*.

**Supplementary Figure**


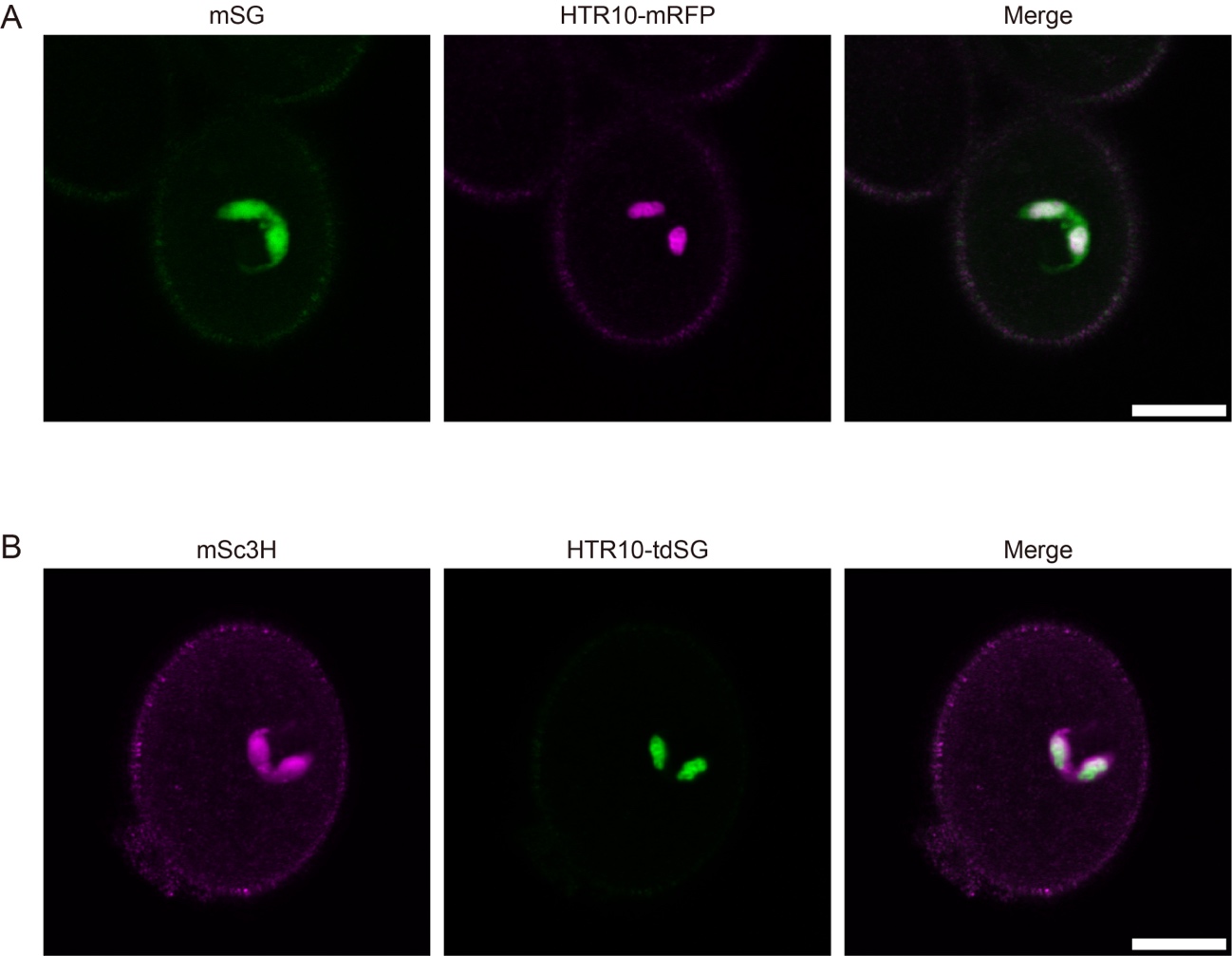


**Supplementary Figure S1. Adaptation of *AHG3* and *P2A*-mediated GESENI Bypassing system to other fluorescent proteins.**

Representative confocal images of mature pollen grains are shown for: (A) *pAHG3:AHG3-P2A-mSG pHTR10:HTR10-mRFP* (Ingoff et al. 2007), (B) *pAHG3:AHG3-P2A-mSc3H pHTR10:HTR10-tdSG.* Scale bars: 10 µm.

References

Ando, R., Shimozono, S., Ago, H. *et al.* (2024) StayGold variants for molecular fusion and membrane-targeting applications. Nat Methods 21, 648–656.

Chen, T.W., Wardill, T.J., Sun, Y., Pulver, S.R., Renninger, S.L., Baohan, A., et al. (2013) Ultrasensitive fluorescent proteins for imaging neuronal activity. Nature. 499: 295-300.

Hirano, M., Ando, R., Shimozono, S. et al. (2022) A highly photostable and bright green fluorescent protein. Nat Biotechnol 40, 1132–1142.

Ingouff M, Hamamura Y, Gourgues M, Higashiyama T, Berger F. (2007) Distinct dynamics of HISTONE3 variants between the two fertilization products in plants. Curr Biol. 19, 1032–1037.

Kimata Y., Higaki T., Kawashima T., Kurihara D., Sato Y., Yamada T., Hasezawa S., Berger F., Higashiyama T., Ueda M. (2016) Cytoskeleton dynamics control the first asymmetric cell division in Arabidopsis zygote. Proc Natl Acad Sci U S A. 113:14157–14162.

Motomura K., Takeuchi H., Notaguchi M., Tsuchi H., Takeda A., Kinoshita T., Higashiyama T., and Maruyama D. (2021) Persistent directional growth capability in Arabidopsis thaliana pollen tubes after nuclear elimination from the apex. Nat Commun 12: 2331.

Nakagawa T, Suzuki T, Murata S, Nakamura S, Hino T, Maeo K, Tabata R, Kawai T, Tanaka K, Niwa Y, et al. (2007) Improved gateway binary vectors: high-performance vectors for creation of fusion constructs in transgenic analysis of plants. Biosci Biotechnol Biochem 71: 2095–2100.

Xiong, H., Chang, Q., Ding, J., Wang, S., Zhang, W., Li, Y., Wu, Y., Lin, P., Yang, C., Liu et al. (2025) A highly stable monomeric red fluorescent protein for advanced microscopy. Nat. Methods 22:1288–1298.
